# Synthesis and Biological Evaluation of Novel Alkyl Amine Substituted Icariside II Derivatives as Potential Anticancer Agents

**DOI:** 10.3390/molecules23092146

**Published:** 2018-08-27

**Authors:** Tong Wu, Ting Li, Ya-Nan Kang, Li Liu, Xi-Man Wang, Jin-Shuai Lan, Yue Ding, Tong Zhang

**Affiliations:** 1 School of Pharmacy, Shanghai University of Traditional Chinese Medicine, Shanghai 201203, China; 13501919930@163.com (T.W.); yanankk1993@outlook.com; (Y.-N.K.) 15216678512@163.com (L.L.); 15000655647@126.com (X.-M.W.); zhangtongshutcm@hotmail.com (T.Z.); 2 Experiment Center for Teaching and Learning, Shanghai University of Traditional Chinese Medicine, Shanghai 201203, China; liting201304@163.com

**Keywords:** icariside II, anticancer, apoptosis, structure-activity relationship

## Abstract

A series of novel alkyl amine-substituted icariside II (ICA II) derivatives were synthesized by Mannich reactions at the 6-C position (compounds **4a**–**d**) and changing the carbon chain length at the 7-OH position (compounds **7a**–**h**), and their in vitro antitumor activity towards human breast cancer lines (MCF-7 and MDA-MB-231) and human hepatoma cell lines (HepG2 and HCCLM3-LUC) were evaluated by the MTT assay. Compared with ICA II, most of the twelve derivatives showed good micromole level activity and a preliminary structure-activity relationship (SAR) for the anticancer activity was obtained. Compound **7g** showed the most potent inhibitory activity for the four cancer cell lines (13.28 μM for HCCLM3-LUC, 3.96 μM for HepG2, 2.44 μM for MCF-7 and 4.21 μM for MDA-MB-231), which was 2.94, 5.54, 12.56 and 7.72-fold stronger than that of ICA II. The preliminary SAR showed that the introduction of a alkyl amine substituent at 6-C was not favorable for the anticancer activity, while most of the 7-*O*-alkylamino derivatives exhibited good antitumor activity and the anticancer activity 7-*O*-alkylamino derivatives were influenced by the alkyl chain length and the different terminal amine substituents. Furthermore, the effects of compound **7g** on apoptosis and cell cycle of MCF-7 cells were further investigated, which showed that compound **7g** triggered apoptosis and arrested the cell cycle at the G0/G1 phase in MCF-7 cells. Our findings indicate that compound **7g** may be a promising anticancer drug candidate lead.

## 1. Introduction

Cancer is one of the major causes of death across the world, owing to its great increasing morbidity and significant mortality [1]. For example, liver cancer has been recognized as the third most common cause of cancer deaths [2]. Breast cancer is one of the common malignant tumors in the female population, ranking first in female malignant tumor mortality [3,4]. Cancer is caused by rapid, uncontrolled, and pathological proliferation of abnormally transformed cells [5]. Chemotherapy is a common method to treat malignancies, but resistance to chemotherapeutic agents, lack of selectivity and serious adverse effects, are the primary challenges of cancer therapy [6,7]. Thus, searching for novel and effective anticancer agents with minimal side effects is considered to be of great value.

**Figure 1:**
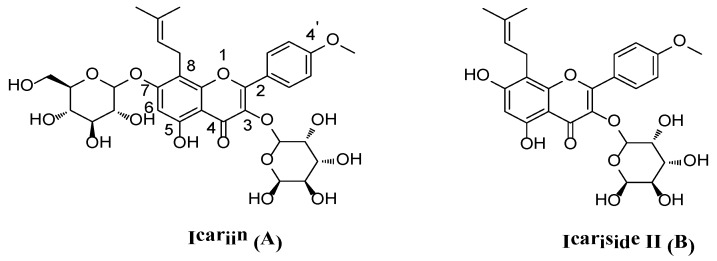
The structures of icariin (**A**) and icariside II (**B**).

With the development of Traditional Chinese Medicine (TCM), it nowadays plays an important role in anti-cancer drug discovery. TCMs can treat cancer through different molecular targets and multiple signaling pathways, such as inducing apoptosis, suppressing proliferation, restraining tumor to transfer, reversing multidrug resistance and enhancing immunity. Moreover, because they cause few adverse effects, more and more TCMs have been applied in clinical studies [8]. Epimedii folium (EF) is the dried leaves from *E. sagittatum* (Sieb.et Zucc.) Maxim., *Epimedium brevicornu* Maxim., *E. koreanum* Nakai, *E. pubescens* Maxim. EF, also named yinyanghuo, barrenwort, copper wire grass and faeries spleen, and has been used as a tonic herb for strengthening the bones and nourishing the kidneys in East Asia, particularly in China, Korea and Japan, for thousands of years [1]. Icariin (ICA), an 8-isopentenyl flavonoid glycoside (Figure 1, compound **A**), is the main active ingredients of EF [9]. ICA possesses a wide-range of pharmacological effects, such as antidepressant-like, hormone regulation, anti-inflammatory, neuroprotective, antioxidative, antirheumatic, and antiosteoporotic effects [10,11,12,13,14]. Moreover, ICA was reported to exhibit anticancer activity against a series of human cancer cell lines. For example, Zheng et al. found that ICA could suppress thyroid carcinoma cell (B-CPAP) proliferation by inducing high expression of intracellular ROS and reducing the expression of antioxidase [15]; ICA could induce apoptosis and arrest cell cycle at S phase in medulloblastoma cells to suppress proliferation [16]. Icariside II (ICA II), another active ingredient of EF (Figure 1, compound **B**), is formed by the intestinal flora from ICA by the loss of the glycosyl moiety at the C-7 position [17]. Our group has worked extensively on the development of ICA and found that 91.2% of ICA was transformed into ICA II after oral administration, and direct oral administration of ICA II increased its concentration in the body leading to faster absorption, slower metabolism and higher absolute bioavailability [18]. Compared with ICA, ICA II possesses stronger activity against inflammation, osteoporosis, cancer, and improves erectile dysfunction [19,20,21,22,23]. We have explored the anticancer activity of ICA II and ICA, and the results (see Table 1) showed that ICA II had better anticancer activities than ICA. Thus, ICA II has attracted increasing research interest. However, because the amount of natural ICA II in EF was about only 0.01% g/g, which is about 1/60–1/6 that of ICA, we also have optimized biotransformation of ICA into ICA II by β-Gglucosidase for further pharmaceutical research [24].

Like many other biologically active flavonoids such as naringenin and quercetin, ICA II is a hydrophobic drug with low absorptive permeability and a high rate of efflux via apical efflux transport [25]. ICA II only showed moderate anticancer activities (see Table 1). Therefore, improving the potency against cancer of ICA II is important for its further application. A literature survey shows that most of the important classes of drugs are nitrogen-containing, and the presence of an amine moiety in drugs may increase their biological potency due to the greater number of molecular sites for electrophilic attack by cellular constituents, as well as due to the cascade effect of preferential chemosensitization [26]. Amine moieties in drugs could also enhance their physicochemical properties (e.g., water solubility) and improve the bioavailability of bioactive molecules [27]. Continuing our study on the chemistry and biology of ICA II, a nitrogen-containing hydrophilic, heterocyclic ring was introduced for the first time at the 6-C position and the 7-OH of ICA II to improve its potency against cancer.

Firstly, we established the hydrolysis process of ICA to get ICA II from our previous reports [24], and a novel series of nitrogen-containing ICA II derivatives were synthesized with a certain length of carbon chain at the 7-OH position and by the Mannich reaction at the 6-C position. Furthermore, we also evaluated the effect of the synthesized derivatives on cell proliferation against human breast cancer cell lines (MCF-7 and MDA-MB-231) and human liver cancer lines (HepG2 and HCCLM3-LUC). Lastly the effects of the most active compound **7g**, on cell cycle and apoptosis of MCF-7 cells were further investigated.

## 2. Results and Discussion

### 2.1. Chemistry

**Scheme 1:**
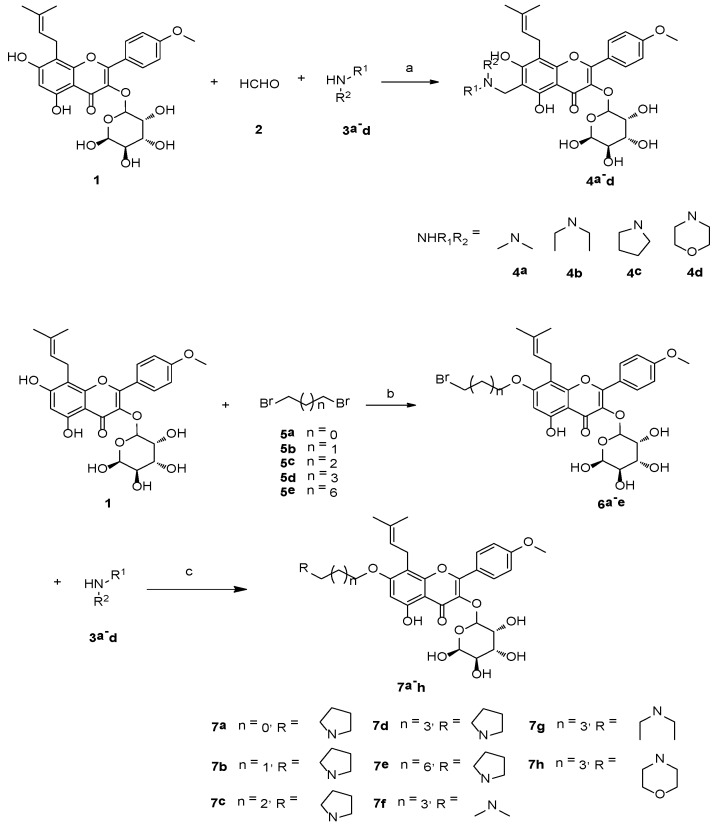
*Reagents and conditions*: (**a**) CH_3_OH, HCl, reflux, 3 h; (**b**) CH_3_CN, K_2_CO_3_, reflux, 12 h; (**c**) CH_3_CN, K_2_CO_3_, reflux, 12 h.

The target nitrogen-containing compounds **4a**–**d** and **7a**–**h** were synthesized as shown in Scheme 1 Compounds **4a**–**d** were synthesized by the Mannich condensation of ICA II (1), formaldehyde (2), and secondary amines **3a**–**d** in a single-step process with good yields. Due to the intramolecular hydrogen bond between 5-OH and 4-ketone, the reactivity of the 5-OH was lower than that of the 7-OH position [28]. Alkylation of ICA II (1) at the 7-OH position with dihalogenated hydrocarbons **5a**–**e** was catalyzed by K_2_CO_3_ to afford the intermediates **6a**–**e** in 50–65% yields. Then nucleophilic substitution between secondary amines and the halogenated hydrocarbons was accomplished to get the final products **7a**–**h** in 70–85% yields. The structures of all synthesized compounds were characterized via ^1^H-NMR, ^13^C-NMR and MS data. In the ^1^H-NMR spectra of compounds **4a**–**d**, a signal at δ 3.8–4.1 indicated the presence of an aminomethyl group at the C-6 of the ICA II scaffold, while in the ^1^H-NMR spectra of compounds **7a**–**h**, the signals at δ 2.5–2.9 and δ 1.6–1.7 indicated the presence of the alkyl amine and the alkyl chain, respectively.

### 2.2. Cell Viability Analysis and Discussion on the Preliminary Structure-Activity Relationship (SAR)

All the novel alkylamino derivatives of ICA II were evaluated for their anti-proliferative activity against human breast cancer cell lines (MCF-7 and MDA-MB-231) and human liver cancer lines (HepG2 and HCCLM3-LUC) by MTT assays. All the cells were incubated with different concentrations of ICA II derivatives for 48 h, and doxorubicin and ICA II were further used as reference compounds. The anticancer activity of the tested compounds was described as the concentration of drug inhibiting 50% cell growth (IC_50_) and is summarized in Table 1. 

**Figure 2:**
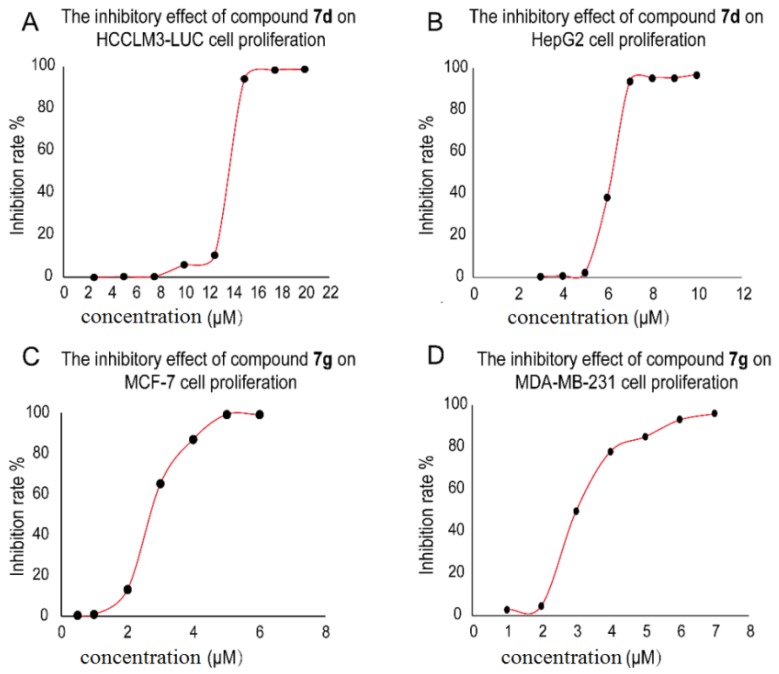
The dose-response curves for MTT assay of compound **7d** (**A**,**B**) on HCCLM3-LUC cells and HepG2 cells, compound **7g** (**C**,**D**) on MCF-7 cells and MDA-MB-231 cells.

The dose-response curves of representative compound **7d** (HCCLM3-LUC cells and HepG2 cells), and compound **7g** (MCF-7 cells and MDA-MB-231 cells) are shown in Figure 2. Among the target compounds, most 7-*O*-alkylamino derivatives **7a**–**h** exhibited comparable or lower IC_50_ values compared with ICA II against all the four cancer cell lines. Namely, these compounds possessed a broad spectrum of anticancer ability, and some compounds possessed IC_50_ values below 10 μM particularly. Compound **7g** showed the most potent inhibitory activity for the four cancer cell lines (13.28 μM for HCCLM3-LUC, 3.96 μM for HepG2, 2.44 μM for MCF-7 and 4.21 μM for MDA-MB-231), which was 2.94, 5.54, 12.56 and 7.72-fold stronger than those of the reference compound ICA II respectively. The Mannich base compounds **4a**–**d** displayed lower cytotoxic activities than ICA II, suggesting that the introduction of an alkyl amine substituent at 6-C was not suitable for their anticancer activity.

For 7-*O*-alkylamino derivatives, to determine the effect of alkyl chain length on activity, compounds **7a**–**e** with different carbon atoms were firstly synthesized. The anti-proliferation activity of compounds for all the four cell lines changed significantly with increasing alkyl chain (CH_2_)_n_ (n from 2 to 8). For example, from the IC_50_ values of **7a**–**e** for MDA-MB-231, compound **7a** (16.85 μM) containing a two carbon spacer had lower inhibitory activity than the other compounds (except for compound **7c**) with longer carbon chain lengths. This suggested that the longer linker length seemed to be favorable for the anticancer activity, but when the alkyl chain length changed from 3 to 8, the anti-proliferation activity of compounds was similar. Based on the above, we chose the alkyl chain with *n* = 3 for further study. With the optimal linker in hand, we planned to introduce different terminal amine substituents to investigate the possible effects on anticancer activity.

With a cyclic amine group (a morpholinyl or pyrrolidyl group) at the terminal position of 7-*O*-alkylamino derivatives, compounds **7d** and **7h** (with IC_50_ at 7.14–15.31 μM) exhibited good inhibition, which were 2.6–2.9 fold (HCCLM3-LUC), 2.9–3.1 fold (HepG2), 2.1–2.8-fold (MCF-7) and 3.2–3.4-fold (MDA-MB-231) more potent than ICA II. Interestingly, it was found that the anticancer activities of compound **7g** containing a *N*,*N*-diethylamino group was higher than that of the *N*-containing heterocyclic compounds **7d** and **7h**. However, the *N*,*N*-methylamino substitution in the compound **7f** (with IC_50_ at 19.92–46.82 μM) exhibited lower activities than substitutions with pyrrolyl (**7d**), *N*,*N*-diethylamino (**7g**) or piperidyl (**7h**) groups, respectively. This may be due to the different volumes of the side chains. In addition, it was worth mentioning that nearly all the derivatives possessed better selectivity against breast cancer cell lines than liver cancer cell lines, although the anticancer activity SAR needs further research.

### 2.3. Cell Morphological Assessment

**Figure 3:**
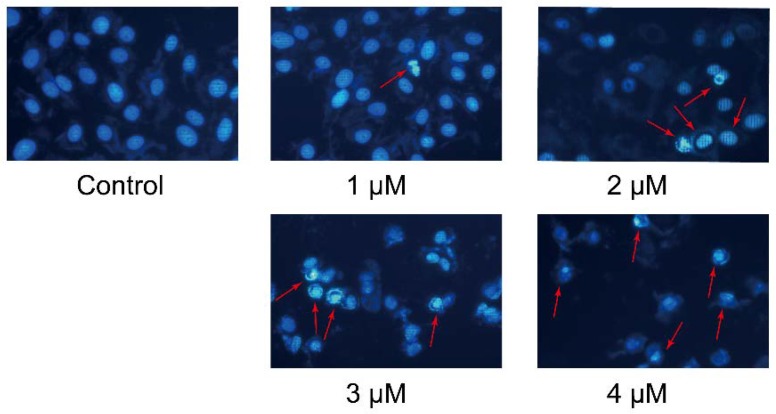
Fluorescent micrographs of Hoechst 33258 stained nuclear patterns when MCF-7 cells treated with compound **7g** at 1–4 μM for 48 h (400×). The red arrows indicate the apoptotic cells.

The anti-proliferative activity of compound **7g** was further confirmed by cell morphological assessment. Apoptosis can induce characteristic morphological changes in cells, such as nuclear shrinkage, DNA degradation, chromatin condensation, apoptotic body formation, cytoskeletal disintegration, which can be observed through fluorescence microscopy after by Hoechst 33258 staining of cell nuclei [29]. MCF-7 cells were exposed to different concentrations of compound **7g** (1–4 μM) for 48 h, and untreated cells served as control. As shown in Figure 3, the nuclei of the cells in the control were round and showed a homogeneous blue fluorescence, whereas the treated cells showed nucleus shrinkage, chromatin condensation and nuclear fragmentation as a strong blue-white fluorescence when treated with different concentrations of compound **7g** for 48 h. Different from intact, round and large nuclei, multiple apoptotic bodies with irregular shapes and sizes appeared in apoptotic cells (red arrows in the figure). In addition, the number of cells with shrunk nucleii was gradually increased with compound **7g** (1–4 μM), and the cytoskeleton was disintegrated at the 3 or 4 μM concentration of compound **7g**. These results all indicated that compound **7g** could induce apoptosis in MCF-7 cells.

### 2.4. Cell Cycle Distribution Analysis

**Figure 4:**
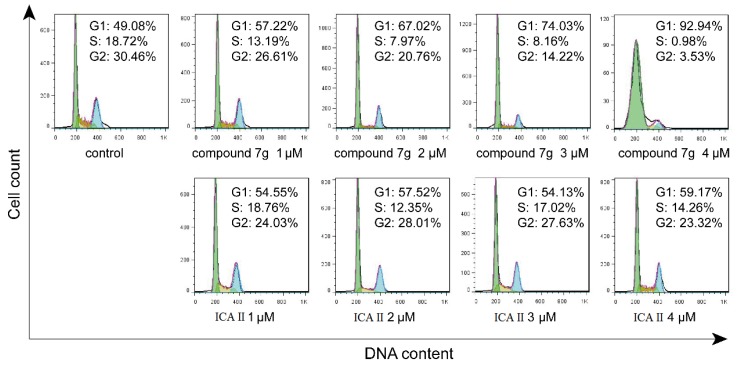
Effect of compound **7g** and ICA II on cell cycle distribution on MCF-7 cells. Cell cycle distribution after treatment with compound **7g** and ICA II at 1–4 μM for 48 h.

The cell cycle is the basic process of cell life activities, and the main characteristic of tumors is that the cell cycle is out of control, which maintains unrestrained cell proliferation and uncontrolled cell division. Therefore, cell cycle arrest has been appreciated as an important route for cancer therapy [30]. To investigate the effect of compound **7g** on the cell cycle of proliferating cancer cells MCF-7 cells were incubated with compound **7g** at different concentrations (1–4 μM) for 48 h, and the resulting cell cycle distribution was analyzed using flow cytometry after propidium iodide (PI) staining. As shown in Figure 4, the percentages of cells at G_2_ phase and S phase were reduced from 26.61% to 3.53% and 13.19% to 0.98%, respectively, when treated with compound **7g** (1–4 μM), as compared with the 30.46% (G_2_ phase) and 18.72% (S phase), respectively, in the control. The percentage of cells in G_0_/G_1_ phase increased from 57.22% to 92.94%. However, no significant changes of the G_0_/G_1_ values (54.55% to 59.17%), G_2_ values (24.03% to 23.32%) and S values (18.76% to 14.26%) obtained were observed with ICA II. The increased percentage of cells at the G_0_/G_1_ phase and the cytotoxic activity suggested that compound **7g**, could arrest cell cycle in the G_0_/G_1_ phase of MCF-7 cells, and compound **7g** demonstrated stronger anticancer activity than ICA II, which agrees with the cell viability analysis results.

### 2.5. Assessment of Apoptotic Analysis

**Figure 5:**
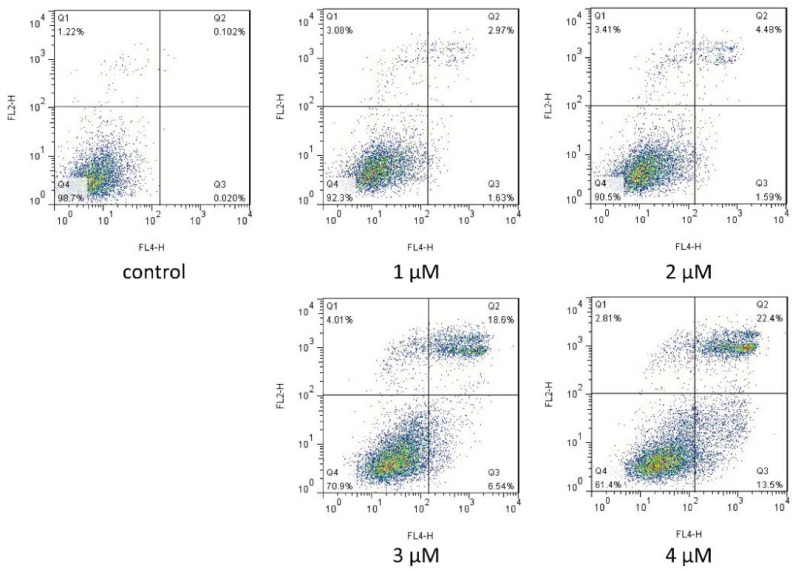
Compound **7g** induced apoptosis in MCF-7 cells. After treatment with compound **7g** at different concentrations (1–4 μM), the apoptotic rates of MCF-7 cells were determined by Annexin V-APC and PI double staining using flow cytometry.

To investigate the bioactivity of compound **7g** against MCF-7 cells, the cells were treated with compound **7g** at different concentrations (1–4 μM). Treated cells were labeled using Annexin V-APC and PI. The rates of cell apoptosis are shown in Figure 5. Treatment with low concentrations of compound **7g** (1 or 2 μM) caused no significant apoptosis compared with control. However, 3 or 4 μM concentrations of compound **7g** induced a high degree of apoptosis in MCF-7 cells at both the early and late stages. The percentage of late apoptotic cells (upper right quadrant) was increased from 2.97% to 22.4%, and the percentage of early apoptotic cells (lower right quadrant) was increased from 1.63% to 13.5%. This result indicated that compound **7g** could induce both early and late apoptosis in MCF-7 cells in a dose-dependent manner, and cytotoxicity to MCF-7 cells occurred at micromolar concentrations.

## 3. Conclusions

In conclusion, various alkyl amide derivatives of ICA II were synthesized and evaluated for their anticancer activity against four different cancer cell lines. Most of the selected compounds possessed good in vitro antitumor activity with a broad spectrum. The SAR showed the most 7-*O*-alkylamino derivatives showed good antitumor activity against all the four cancer cell lines, while the Mannich base compounds displayed lower cytotoxic activities. Moreover, the optimal length of the alkyl chain and different terminal amine substituents of 7-*O*-alkylamino derivatives markedly affected the antitumor activities. Among the target compounds, compound **7g** exhibited the most significant anti-proliferative activity for the four tested cancer cell lines (13.28 μM for HCCLM3-LUC, 3.96 μM for HepG2, 2.44 μM for MCF-7 and 4.21 μM for MDA-MB-231). Compound **7g** induction of MCF-7 cells apoptosis was confirmed in an Annexin V-APC/PI staining apoptotic assay and Hoechst 33258 staining analysis. In the cell cycle distribution, compound **7g** could arrest the cell population at the G_0_/G_1_ phase. The results indicated that **7g** exhibited enhanced anticancer activity against human breast cancer cells compared with ICA II. Thus, the novel 7-*O*-alkylamino derivative of ICA II, compound **7g**, could be a promising antitumor agent for breast cancer and is worthy of further development.

## 4. Experimental Section

### 4.1. Materials and Methods

All chemicals (reagent grade) used were purchased from Sino Pharm Chemical Reagent Co., Ltd. (Shanghai, China). Reaction progress was monitored using analytical thin layer chromatography (TLC) on precoated silica gel GF254 (Qingdao Haiyang Chemical Plant, Qingdao, China) plates and the spots were detected under UV light (254 nm). Melting point was measured on an XT-4 micromelting point instrument (Agilent, Shanghai, China) and are uncorrected. ^1^H-NMR (400 MHz) and ^13^C-NMR spectra (100 MHz) were measured on an AVANCE III spectrometer (Bruker, Germany) at 25 °C and referenced to TMS. Chemical shifts are reported in ppm (δ) using the residual solvent line as internal standard. Splitting patterns are designed as s, singlet; d, doublet; t, triplet; m, multiplet. Mass spectra were obtained on a MS Agilent 1100 Series LC/MSD Trap mass spectrometer (ESI-MS, Agilent, China).

### 4.2. General Experimental Procedure for Mannich Base Derivatives ***4a**–**d***

A mixture of 37% aqueous formaldehyde (1.8 mL, 0.22 mmol) and secondary amine (0.22 mmol) in 10 mL of methanol and 0.02 mL of 15% HCl (aq) was stirred at 80 °C until complete homogenization. The solution obtained was added slowly to a solution of icariside II (98 mg, 0.19 mmol) in methanol, and the reaction mixture was refluxed for 1–3 h. After the reaction was complete, the mixture was rotary evaporated to distill off the methanol, then water was added, and the aqueous phase was extracted three times with EtOAc and dried over anhydrous Na_2_SO_4_. The solvent was removed under the reduced pressure. The residue was crystallized from EtOAc/petroleum ether (PE) to afford compounds **4a**–**d** as yellow crystals in 70–85% yields.

*6-((Dimethylamino)methyl)-5,7-dihydroxy-2-(4-methoxyphenyl)-8-(3-methylbut-2-en-1-yl)-3-(((3R,4R,5R,6S)-3,4,5,6-tetrahydroxytetrahydro-2H-pyran-2-yl)oxy)-4H-chromen-4-one* (**4a**). Yield 70%; yellow solid; m.p. 80.0–81.8 °C; ESI/MS *m*/*z*: 574.2 [M + H]^+^; ^1^H-NMR (400 MHz, CDCl_3_) δ 12.74 (s, 1H), 7.84 (d, *J* = 8.7 Hz, 2H), 7.02 (d, *J* = 8.8 Hz, 2H), 5.50 (s, 1H), 5.32 (s, 1H), 5.22 (s, 1H), 3.88 (s, 3H), 3.84 (s, 2H), 3.62 (m, 7H), 3.48–3.42 (m, 2H), 3.31 (dd, *J* = 13.7, 7.6 Hz, 1H), 2.41 (s, 6H), 1.76 (s, 3H), 1.70 (s, 3H). ^13^C-NMR (100 MHz, CDCl_3_) δ 178.4, 164.1, 161.7, 157.1, 156.7, 153.8, 134.9, 131.8, 130.6, 130.6, 123.0, 122.6, 114.0, 114.0, 106.5, 104.2, 103.4, 101.7, 72.5, 71.5, 70.60, 70.3, 55.5, 50.9, 44.1, 44.1, 25.8, 18.0, 17.1.

*6-((Diethylamino)methyl)-5,7-dihydroxy-2-(4-methoxyphenyl)-8-(3-methylbut-2-en-1-yl)-3-(((3R,4R,5R,6S)-3,4,5,6-tetrahydroxytetrahydro-2H-pyran-2-yl)oxy)-4H-chromen-4-one* (**4b**). Yield 79%; yellow solid; m.p. 155.0–157.1 °C; ESI/MS *m*/*z*: 602.3 [M + H]^+^; ^1^H-NMR (400 MHz, CDCl_3_) δ 12.75 (s, 1H), 7.85 (d, *J* = 8.7 Hz, 2H), 7.02 (d, *J* = 8.7 Hz, 2H), 5.50 (s, 1H), 5.24 (s, 1H), 4.46 (s, 1H), 3.94 (m, 2H), 3.91 (m, 6H), 3.88 (s, 3H), 3.46 (m, 3H), 3.35–3.24 (m, 1H), 2.73 (dd, *J* = 14.0, 6.9 Hz, 4H), 1.75 (s, 3H), 1.70 (s, 3H), 1.17 (t, *J* = 7.1 Hz, 6H). ^13^C-NMR (100 MHz, CDCl_3_) δ 178.3, 161.9, 161.6, 157.0, 156.6, 153.8, 134.8, 131.6, 130.6, 130.6, 123.1, 122.7, 113.9, 113.9, 106.6, 103.4, 103.5, 101.6, 72.5, 71.6, 70.6, 70.2, 55.5, 49.1, 46.4, 46.4, 25.8, 18.0, 17.1, 10.8, 10.8.

*5,7-Dihydroxy-2-(4-methoxyphenyl)-8-(3-methylbut-2-en-1-yl)-6-(pyrrolidin-1-ylmethyl)-3-(((3R,4R,5R,6S)-3,4,5,6-tetrahydroxytetrahydro-2H-pyran-2-yl)oxy)-4H-chromen-4-one* (**4c**). Yield 85%; yellow solid; m.p. 115.7–116.5 °C; ESI/MS *m*/*z*: 600.2 [M + H]^+^; ^1^H-NMR (400 MHz, CDCl_3_) δ 12.77 (s, 1H), 7.85 (d, *J* = 8.7 Hz, 2H), 7.02 (d, *J* = 8.8 Hz, 2H), 5.51 (s, 1H), 5.23 (s, 1H), 4.45 (s, 1H), 4.02 (s, 2H), 3.89 (s, 3H), 3.46 (t, *J* = 6.8 Hz, 4H), 3.32 (m, 6H), 2.78 (m, 4H), 1.93 (s, 4H), 1.76 (s, 3H), 1.71 (s, 3H). ^13^C-NMR (100 MHz, CDCl_3_) δ 178.4, 164.7, 161.6, 157.0, 156.3, 153.8, 134.8, 131.7, 130.6, 130.6, 123.1, 122.7, 113.9, 113.9, 106.6, 104.0, 104.0, 101.6, 72.6, 71.6, 70.6, 70.2, 55.5, 53.4, 53.4, 50.8, 25.8, 23.7, 23.7, 18.0, 17.1.

*5,7-Dihydroxy-2-(4-methoxyphenyl)-8-(3-methylbut-2-en-1-yl)-6-(morpholinomethyl)-3-(((3R,4R,5R,6S)-3,4,5,6-tetrahydroxytetrahydro-2H-pyran-2-yl)oxy)-4H-chromen-4-one* (**4d**). Yield 82%; yellow solid; m.p. 155.6–158.7 °C; ESI/MS *m*/*z*: 616.3 [M + H]^+^; ^1^H-NMR (400 MHz, CDCl_3_) δ 12.77 (s, 1H), 7.85 (d, *J* = 8.8 Hz, 2H), 7.03 (d, *J* = 8.8 Hz, 2H), 5.50 (s, 1H), 5.22 (d, *J* = 6.8 Hz, 1H), 4.46 (s, 1H), 3.88 (s, 3H), 3.87 (m, 3H), 3.79 (m, 3H), 3.74–3.69 (m, 2H), 3.52–3.41 (m, 5H), 3.38–3.27 (m, 2H), 2.74–2.63 (m, 3H), 2.53 (s, 2H), 1.76 (s, 3H), 1.71 (s, 3H). ^13^C-NMR (100 MHz, CDCl_3_) δ 178.5, 163.1, 161.7, 157.4, 156.9, 153.7, 134.9, 131.9, 130.7, 130.7, 122.9, 122.4, 114.0, 114.0, 106.5, 104.5, 102.7, 101.7, 72.5, 71.5, 70.6, 70.3, 66.7, 66.7, 55.5, 53.5, 52.7, 52.7, 25.8, 18.0, 17.1.

### 4.3. General Experimental Procedure for the 7-O-Alkylamino Derivatives ***7a**–**h***

Excess potassium carbonate was added as the catalyst to a solution of the ICA II (0.50 mmol, 1 equiv) in acetonitrile (10 mL). After stirring for 10 min, di-halogenated hydrocarbon (2.50 mmol, 5 equiv) was added, and the resulting mixture was stirred at 80 °C for 12 h. The reaction was monitored by thin-layer chromatography. After completion of the reaction, the solid was filtered, and the solution was concentrated in vacuo. The crude product was purified by chromatography on silica gel (PE/EtOAc) to give compounds **6a**–**e** with 50–65% yields. Excess potassium carbonate was added as the catalyst to compounds **6a**–**e** (0.20 mmol, 1 equiv) in acetonitrile (10 mL). After stirring for 10 min, the desired secondary amine (0.40 mmol, 2 equiv) was added, and the reaction mixture was stirred at 80 °C for 12 h. The solid was removed by filtration, and the solution was concentrated *in vacuo*. The crude product was purified by chromatography on silica gel (PE/EtOAc) to give the compounds **7a**–**h** as yellow solid in 55–75% yields.

*5-Hydroxy-2-(4-methoxyphenyl)-8-(3-methylbut-2-en-1-yl)-7-(2-(pyrrolidin-1-yl)ethoxy)-3-(((3R,4R,5R,6S)-3,4,5,6-tetrahydroxytetrahydro-2H-pyran-2-yl)oxy)-4H-chromen-4-one* (**7a**). Yield 60%; yellow solid; m.p. > 250 °C; ESI/MS *m*/*z*: 614.2 [M + H]^+^; ^1^H-NMR (400 MHz, CDCl_3_) δ 12.54 (s, 1H), 7.81 (d, *J* = 8.3 Hz, 2H), 7.00 (d, *J* = 8.2 Hz, 2H), 5.47 (s, 1H), 5.11 (s, 1H), 4.46 (s, 1H), 4.30 (s, 2H), 3.86 (s, 3H), 3.37 (m, 7H), 3.15 (s, 3H), 2.88 (m, 6H), 1.92 (s, 4H), 1.72 (s, 3H), 1.67 (s, 3H). ^13^C-NMR (100 MHz, CDCl_3_) δ 178.6, 161.8, 161.8, 160.2, 157.5, 153.6, 135.1, 131.8, 130.7, 130.7, 122.7, 122.4, 122.4, 114.0, 114.0, 107.8, 105.7, 101.9, 95.7, 72.4, 71.5, 70.4, 67.0, 55.5, 54.8, 54.8, 50.8, 25.7, 23.5, 23.5, 18.1, 17.2.

*5-Hydroxy-2-(4-methoxyphenyl)-8-(3-methylbut-2-en-1-yl)-7-(3-(pyrrolidin-1-yl)propoxy)-3-(((3R,4R,5R,6S)-3,4,5,6-tetrahydroxytetrahydro-2H-pyran-2-yl)oxy)-4H-chromen-4-one* (**7b**). Yield 55%; yellow solid; m.p. 70.8–71.3 °C; ESI/MS *m*/*z*: 628.3 [M + H]^+^; ^1^H-NMR (400 MHz, DMSO-*d*_6_) δ 12.65 (s, 1H), 7.87 (d, J = 8.9 Hz, 2H), 7.14 (d, *J* = 8.9 Hz, 2H), 5.27 (s, 1H), 5.13 (s, 1H), 4.16 (d, *J* = 5.6 Hz, 2H), 4.00 (s, 1H), 3.86 (s, 3H), 3.48 (m, 9.3 Hz, 10H), 2.69 (s, 3H), 2.60 (s, 4H), 1.98 (d, *J* = 6.3 Hz, 2H), 1.74 (s, 3H), 1.69 (s, 3H), 1.62 (s, 3H). ^13^C-NMR (100 MHz, DMSO-*d*_6_) δ 178.7, 165.4, 162.30 161.9, 161.0, 160.1, 157.6, 134.9, 131.7, 131.0, 131.0, 122.8, 122.5, 114.6, 114.6, 107.6, 105.0, 102.5, 96.3, 71.6, 71.2, 70.8, 70.5, 56.0, 53.9, 53.9, 52.4, 28.0, 25.9, 23.5, 21.7, 18.3, 17.9.

*5-Hydroxy-2-(4-methoxyphenyl)-8-(3-methylbut-2-en-1-yl)-7-(4-(pyrrolidin-1-yl)butoxy)-3-(((3R,4R,5R,6S)-3,4,5,6-tetrahydroxytetrahydro-2H-pyran-2-yl)oxy)-4H-chromen-4-one* (**7c**). Yield 70%; yellow solid; m.p. 92.7–94.2 °C; ESI/MS *m*/*z*: 642.3 [M]^+^; ^1^H-NMR (400 MHz, DMSO-*d*_6_) δ 12.51 (s, 1H), 7.85 (dd, *J* = 17.7, 8.9 Hz, 2H), 7.16–7.08 (m, 2H), 5.28 (s, 1H), 5.16 (dd, *J* = 13.3, 6.7 Hz, 1H), 4.12 (t, *J* = 6.0 Hz, 1H), 4.00 (s, 1H), 3.85 (d, *J* = 3.4 Hz, 4H), 3.36 (m, 10H), 2.46–2.38 (m, 3H), 2.15–1.98 (m, 6H), 1.79 (dd, *J* = 14.1, 6.0 Hz, 1H), 1.67 (d, *J* = 3.6 Hz, 5H), 1.62 (s, 5H). ^13^C-NMR (100 MHz, DMSO-*d*_6_) δ 177.6, 166.4, 161.5, 159.4, 156.1, 154.3, 130.9, 130.7, 130.7, 123.6, 123.3, 122.5, 114.6, 114.4, 114.4, 106.5, 102.3, 100.4, 99.8, 71.6, 70.8, 70.6, 69.0, 62.3, 62.3, 55.9, 54.0, 25.9, 25.3, 23.5, 22.0, 21.8, 18.3, 17.9.

*5-Hydroxy-2-(4-methoxyphenyl)-8-(3-methylbut-2-en-1-yl)-7-((5-(pyrrolidin-1-yl)pentyl)oxy)-3-(((3R,4R,5R,6S)-3,4,5,6-tetrahydroxytetrahydro-2H-pyran-2-yl)oxy)-4H-chromen-4-one* (**7d**). Yield 75%; yellow solid; m.p. 140.4–142.9 °C; ESI/MS *m*/*z*: 656.3 [M + H]^+^; ^1^H-NMR (400 MHz, DMSO-*d*_6_) δ 12.54 (s, 1H), 7.87 (d, J = 8.4 Hz, 2H), 7.12 (d, J = 8.6 Hz, 2H), 5.29 (s, 1H), 5.13 (s, 1H), 4.06 (s, 3H), 3.85 (s, 4H), 3.50 (m, 10H), 2.44–2.31 (m, 5H), 1.72 (s, 7H), 1.68 (s, 3H), 1.65 (s, 3H), 1.62 (s, 3H). ^13^C-NMR (100 MHz, DMSO-*d*_6_) δ 175.7, 165.2, 162.2, 161.7, 160.8, 157.2, 150.8, 135.4, 131.5, 130.8, 122.8, 122.0, 114.6, 114.6, 106.3, 102.7, 100.0, 97.5, 95.8, 72.4, 70.8, 70.6, 69.0, 56.1, 55.9, 54.1, 54.1, 28.6, 25.9, 24.8, 24.1, 23.5, 21.7, 18.2, 18.0.

*5-Hydroxy-2-(4-methoxyphenyl)-8-(3-methylbut-2-en-1-yl)-7-((8-(pyrrolidin-1-yl)octyl)oxy)-3-(((3R,4R,5R,6S)-3,4,5,6-tetrahydroxytetrahydro-2H-pyran-2-yl)oxy)-4H-chromen-4-one* (**7e**). Yield 63%; yellow solid; m.p. 139.4–141.4 °C; ESI/MS *m*/*z*: 670.3 [M + H]^+^; ^1^H-NMR (400 MHz, DMSO-*d*_6_) δ 12.65 (s, 1H), 7.87 (d, *J* = 8.9 Hz, 2H), 7.13 (d, *J* = 9.0 Hz, 2H), 5.28 (d, *J* = 1.2 Hz, 1H), 5.13 (t, *J* = 7.0 Hz, 1H), 4.10 (t, *J* = 6.1 Hz, 2H), 4.00 (d, *J* = 1.4 Hz, 1H), 3.86 (s, 3H), 3.49 (m, 10H), 2.69 (s, 4H), 2.60 (d, *J* = 5.9 Hz, 2H), 1.74 (s, 7H), 1.68 (s, 3H), 1.62 (s, 3H), 1.47 (dd, *J* = 14.1, 7.1 Hz, 4H), 1.30 (s, 5H). ^13^C-NMR (100 MHz, DMSO-*d*_6_) δ 178.7, 162.4, 161.8, 160.1, 157.6, 153.3, 134.9, 131.6, 130.9, 130.9, 122.8, 122.5, 122.5, 114.6, 114.6, 107.5, 105.0, 102.5, 96.2, 71.6, 71.2, 70.8, 70.5, 69.1, 56.0, 55.5, 53.7, 29.2, 29.0, 29.0, 27.6, 27.1, 25.9, 25.9, 23.4, 21.7, 18.2, 17.9.

*7-((5-(Dimethylamino)pentyl)oxy)-5-hydroxy-2-(4-methoxyphenyl)-8-(3-methylbut-2-en-1-yl)-3-(((3R,4R,5R,6S)-3,4,5,6-tetrahydroxytetrahydro-2H-pyran-2-yl)oxy)-4H-chromen-4-one* (**7f**). Yield 72%; yellow solid; m.p. 164.6–166.8 °C; ESI/MS m/z: 630.3 [M + H]^+^; ^1^H-NMR (400 MHz, CDCl_3_) δ 12.47 (s, 1H), 7.85 (d, *J* = 8.8 Hz, 2H), 7.02 (d, *J* = 8.9 Hz, 2H), 6.34 (s, 1H), 5.50 (s, 1H), 5.15 (t, *J* = 6.8 Hz, 1H), 4.47 (s, 1H), 4.03 (t, *J* = 6.3 Hz, 2H), 3.88 (s, 3H), 3.79–3.72 (m, 4H), 3.52–3.39 (m, 4H), 3.33 (dd, *J* = 9.4, 6.2 Hz, 1H), 2.43 (s, 6H), 2.44–2.37 (m, 2H), 1.90–1.83 (m, 2H), 1.73 (s, 3H), 1.68 (s, 3H), 1.60 (d, *J* = 7.2 Hz, 2H), 1.53 (d, *J* = 6.5 Hz, 2H). ^13^C-NMR (100 MHz, CDCl_3_) δ 178.7, 162.3, 161.8, 160.2, 157.6, 153.6, 135.0, 131.6, 130.7, 130.7, 122.8, 122.4, 118.2, 114.0, 114.0, 107.8, 105.4, 101.8, 95.6, 72.5, 71.5, 70.3, 66.9, 58.9, 55.5, 53.7, 53.7, 29.0, 26.2, 25.8, 23.8, 18.0, 17.1.

*7-((5-(Diethylamino)pentyl)oxy)-5-hydroxy-2-(4-methoxyphenyl)-8-(3-methylbut-2-en-1-yl)-3-(((3R,4R,5R,6S)-3,4,5,6-tetrahydroxytetrahydro-2H-pyran-2-yl)oxy)-4H-chromen-4-one* (**7g**). Yield 64%; yellow solid; m.p. 134.3–135.9 °C; ESI/MS *m*/*z*: 658.2 [M + H]^+^; ^1^H-NMR (400 MHz, CDCl_3_) δ 12.53 (s, 1H), 7.84 (d, *J* = 8.7 Hz, 2H), 7.02 (d, *J* = 8.7 Hz, 2H), 6.33 (s, 1H), 5.50 (s, 1H), 5.11 (s, 1H), 4.43 (s, 1H), 4.04 (s, 2H), 3.90 (s, 3H), 3.57–3.28 (m, 8H), 3.18 (d, *J* = 7.0 Hz, 5H), 3.06 (s, 2H), 1.92 (s, 4H), 1.71 (s, 3H), 1.68 (s, 3H), 1.61 (s, 2H), 1.41 (t, *J* = 7.1 Hz, 6H). ^13^C-NMR (100 MHz, CDCl_3_) δ 178.6, 161.9, 161.8, 160.2, 157.5, 153.5, 135.0, 132.8, 130.7, 130.7, 122.8, 122.4, 121.9, 114.0, 114.0, 107.7, 104.2, 101.7, 95.6, 72.6, 70.6, 70.3, 67.5, 55.5, 51.3, 50.9, 46.4, 28.7, 25.8, 23.5, 23.3, 18.0, 17.1, 8.6, 8.6.

*5-Hydroxy-2-(4-methoxyphenyl)-8-(3-methylbut-2-en-1-yl)-7-((5-morpholinopentyl)oxy)-3-(((3R,4R,5R,6S)-3,4,5,6-tetrahydroxytetrahydro-2H-pyran-2-yl)oxy)-4H-chromen-4-one* (**7h**). Yield 71%; yellow solid; m.p. 227.2–229.5 °C; ESI/MS *m*/*z*: 672.2 [M + H]^+^; ^1^H-NMR (400 MHz, DMSO-*d*_6_) δ 12.66 (s, 1H), 7.87 (d, *J* = 8.9 Hz, 2H), 7.14 (d, *J* = 9.0 Hz, 2H), 5.14 (t, *J* = 7.0 Hz, 1H), 5.01 (d, *J* = 4.3 Hz, 1H), 4.13 (t, *J* = 6.2 Hz, 2H), 4.00 (s, 1H), 3.86 (s, 3H), 3.37–3.34 (m, 10H), 3.19–3.02 (m, 4H), 2.77 (s, 6H), 1.79 (dd, *J* = 14.1, 6.6 Hz, 2H), 1.68 (d, *J* = 12.0 Hz, 5H), 1.63 (s, 3H), 1.52–1.42 (m, 2H). ^13^C-NMR (100 MHz, DMSO-*d*_6_) δ 178.7, 162.3, 161.9, 160.1, 157.6, 153.4, 134.9, 131.7, 131.0, 131.0, 122.8, 122.5, 114.6, 114.6, 107.5, 105.0, 102.5, 96.3, 71.6, 71.2, 70.8, 70.5, 68.7, 56.9, 56.9, 56.0, 56.0, 42.6, 42.6, 28.5, 26.0, 23.8, 22.9, 18.3, 17.9.

### 4.4. Cell Lines and Cell Culture Conditions

All cells were obtained from the American Type Culture Collection (ATCC, Manassas, VA, USA). Human breast cancer cells (MCF-7 and MDA-MB-231) and human hepatocellular carcinoma cell lines (HCCLM3-LUC) were cultured in DMEM (HyClone, Logan, UT, USA) medium, supplemented with 10% fetal bovine serum (FBS, Gibco, Life Technologies, New York, NY, USA) and antibiotics (100 mg/mL streptomycin and 100 units/mL penicillin (Gibco, Life Technologies). Human liver carcinoma cell lines (HepG2) were cultured in MEM (HyClone) medium, supplemented with 10% fetal bovine serum (FBS) and antibiotics. Human cancer cells were maintained in humidified atmosphere with 5% CO_2_ and 95% air at 37 °C in a carbon dioxide incubator (SANYO, CO_2_ incubator, Osaka, Japan).

### 4.5. Cell Viability Inhibition Assay

The cytotoxicity of compounds **4a**–**d** and **7a**–**g** formulation against human breast cancer cells (MCF-7 and MDA-MB-231) and human hepatic carcinoma cells (HepG2 and HCCLM3-LUC) were evaluated by measuring the cell viability with CCK-8 kit (Beyotime Biotechnology Co., Ltd., Shanghai, China). Four cancer cells were seeded into 96-well plates at 5 × 10^3^ cells per well in 100 µL RPMI 1640 medium at 37 °C under a 5% CO_2_ atmosphere for 12 h, after cancer cells were treated with various concentrations of ICA II derivatives from 0 to 100 μM for 48 h. For comparison purpose, doxorubicin, and ICA II were used as reference compounds. 100 µL free medium containing 10 µL CCK-8 was added to each well according to the manufacturer’s protocol and cells were incubated for a further 1 h. Then, the absorbance was measured using a micro-plate reader (ThermoFisher, Varioskan Flash, Finland) at 450 nm wavelength. Untreated cells in the wells were used as blank controls. The results are shown as the average cell viability = ([OD_treat_ − OD_blank_]/[OD_control_ − OD_blank_] × 100%). The cytotoxic activity was expressed as the half maximal inhibitory concentration (IC_50_) value [22,23]. Every sample was repeated in triplicate wells.

### 4.6. Cell Morphological Assessment

To detect morphological evidence of apoptosis, cells were visualized following DNA staining with the fluorescent dye Hoechst 33,258. The MCF-7 cells were plated in glass-bottom culture dish at a density of 1 × 10^4^ cells and were incubated overnight. The cells were treated with 1–4 μM compound **7g** for 48 h. The cells of each dish were stained with Hoechst 33,258 (Beyotime Biotechnology Co., Ltd., Shanghai, China) in a dark room for 5 min, the cells were washed with PBS and then were observed by a fluorescent microscope (Axio Observer A1, Zeiss, Japan).

### 4.7. Cell Cycle Distribution Analysis

To investigate the effect of the most promising compound **7g** on cell division cycle of proliferating cancer cells MCF-7 cells were plated at 1 × 10^6^ cells per 6 cm dish and cultured overnight. The cells were incubated with compound **7g** at 1–4 μM for 48 h. The cells of each well were harvested, washed twice with PBS and fixed with 70% EtOH at 4 °C overnight. Then, the fixed cells were washed twice with PBS to removed EtOH and harvested. The cells were resuspended in 500 μL of staining buffer containing propidium iodide (PI) and RNase. The mixture was incubated in a dark room for 30 min at 37 °C, then analyzed using a FACS XCalibur flow cytometer (Becton Dickinson, San Jose, CA, USA) and the cell cycle analyzed using the FlowJo 7.6 software (Tree Star, Ashland, OR, USA).

### 4.8. Assessment of Apoptotic Analysis

The MCF-7 cells were plated at the concentration of 1 × 10^6^ cells into 6-well plates. After overnight growth, cells were treated with 1–4 μM compound **7g** for 48 h. The cells of each dish were trypsinized, washed twice with PBS. The cells were resuspended in 500 μL of binding buffer containing propidium iodide (PI) and Annexin-V-APC, and incubated in a dark room for 30 min at room temperature, then subjected to cell apoptotic analysis using a FACSCalibur flow cytometer. Annexin V-APC and PI emission were detected in the FL4 and FL2 channels of flow cytometry, respectively. Cells in the lower left quadrant, lower right quadrant, upper right quadrant represent normal cells, early apoptosis and late apoptosis or necrosis, respectively. The data were analyzed using the Flowjo 7.6 software.

## Figures and Tables

**Table 1:** Antitumor cell proliferation activity of alkyl amine derivatives of ICA II by MTT assay.

Compound	IC_50_ (μM) ^a^			
	HCCLM3-LUC	HepG2	MCF-7	MDA-MB-231
**4a**	>50	>50	>50	>50
**4b**	>50	>50	>50	>50
**4c**	>50	>50	>50	>50
**4d**	>50	>50	>50	>50
**7a**	18.72 ± 0.33	15.41 ± 0.60	14.97 ± 0.12	16.85 ± 0.24
**7b**	14.23 ± 1.02	7.67 ± 0.83	10.53 ± 1.34	8.42 ± 0.45
**7c**	Nd ^b^	Nd	Nd	Nd
**7d**	13.51 ± 0.93	7.67 ± 0.67	10.98 ± 0.22	10.15 ± 0.74
**7e**	13.9 ± 1.12	7.12 ± 0.83	10.94 ± 0.94	8.58 ± 0.69
**7f**	46.82 ± 5.94	19.92 ± 1.19	31.46 ± 2.37	31.08 ± 4.62
**7g**	13.28 ±0.44	3.96 ± 0.37	2.44 ± 0.18	4.21 ± 0.31
**7h**	15.31 ± 0.87	7.14 ± 0.69	14.61 ± 1.05	9.57 ± 0.59
**Icariin**	>100	>100	>100	>100
**Icariside II**	39.04 ± 1.92	21.92 ± 2.04	30.64 ± 3.46	32.53 ± 2.18
**doxorubicin**	1.97 ± 0.11	0.96 ± 0.06	16.16 ± 0.47	2.36 ± 0.14

^a^: Compound concentration required to inhibit the rate by 50%. Data are expressed as mean ± SD from the dose–response curves of 3–5 independent experiments. ^b^: Nd means not detected due to the poor solubility.
